# Peripheral T-cell responses of EphB2- and EphB3-deficient mice in a model of collagen-induced arthritis

**DOI:** 10.1007/s00018-024-05197-0

**Published:** 2024-04-01

**Authors:** Sara Montero-Herradón, Javier García-Ceca, Marta Villarejo-Torres, Agustín G. Zapata

**Affiliations:** 1 Department of Cell Biology, Faculty of Biological Sciences, Complutense University of Madrid, 28040 Madrid, Spain; 2 Health Research Institute, Hospital 12 de Octubre (imas12), 28041 Madrid, Spain

**Keywords:** Eph/ephrin-B, T-lymphocytes, T-cell selection, Arthritis

## Abstract

Both EphB2- and EphB3-deficient mice exhibit profound histological alterations in the thymic epithelial network but few changes in T-cell differentiation, suggesting that this organization would be sufficient to produce functional T lymphocytes. Also, other antigen-presenting cells involved in immunological education could substitute the thymic epithelium. Accordingly, we found an increased frequency of plasmacytoid dendritic cells but not of conventional dendritic cells, medullary fibroblasts or intrathymic B lymphocytes. In addition, there are no lymphoid infiltrates in the organs of mutant mice nor do they contain circulating autoantibodies. Furthermore, attempts to induce arthritic lesions after chicken type II collagen administration fail totally in EphB2-deficient mice whereas all WT and half of the immunized EphB3^−/−^ mice develop a typical collagen-induced arthritis. Our results point out that Th17 cells, IL4-producing Th2 cells and regulatory T cells are key for the induction of disease, but mutant mice appear to have deficits in T cell activation or cell migration properties. EphB2^−/−^ T cells show reduced in vitro proliferative responses to anti-CD3/anti-CD28 antibodies, produce low levels of anti-type II collagen antibodies, and exhibit low proportions of T follicular helper cells. On the contrary, EphB3^−/−^ lymph node cells respond accurately to the different immune stimuli although in lower levels than WT cells but show a significantly reduced migration in in vitro transwell assays, suggesting that no sufficient type II collagen-dependent activated lymphoid cells reached the joints, resulting in reduced arthritic lesions.

**Supplementary Information:**

The online version contains supplementary material available at 10.1007/s00018-024-05197-0.

## Introduction

Eph are the most frequent tyrosine kinase receptors in animal cells. Together with their ligands, ephrins, are involved in numerous cellular processes, such as cell migration, development, tissue homeostasis, etc. [1, 2]. In the immune system, Eph/ephrin signaling acts as a co-receptor of T-cells and/or regulates lymphocyte migration [2].

For many years, we have studied the role of Eph and ephrins in thymus biology, using deficient mice for these molecules or chimeric murine models, concluding that EphB2 and EphB3, together with ephrin-B1 and ephrin-B2, regulate the homeostasis of thymic epithelial cells (TECs), an essential process for the correct maturation of functional T lymphocytes [3]. However, EphB2- and EphB3-deficient mice have quite normal T cell differentiation, including those T cells phenotypically defined by the expression of CD4/CD8 cell markers, positive and negatively selected T-cells or regulatory T (Treg) lymphocytes [4, 5]. In support of this, other studies [6–11] also indicate that, despite profound alterations in the thymic epithelial network key for the acquisition of immune central tolerance, the proportions of distinct T-cell subsets do not vary significantly.

These results suggest that just a few histologically well-organized thymic epithelial areas remaining in the EphB-mutant thymuses would be sufficient for their correct functioning [12]. The altered thymic epithelium could also be replaced functionally by other antigen-presenting cells (APCs) [11] known to be involved in thymic selection, such as B lymphocytes, medullary fibroblasts (mFBs) and/or dendritic cells (DCs) [13–15]. On the other hand, the lack of important phenotypical changes in mutant thymocytes does not ensure a correct function of the distinct T-cell subsets, particularly of those involved in maintaining immune tolerance, although pioneer studies by our group did not find immunological deficits or changes in the TCR repertoire in either the thymus or lymph nodes of EphB-deficient mice [16]. In fact, mutant mice deficient in diverse Eph or ephrins, but not in EphB2 or EphB3, develop autoimmunity [2], although at a low incidence, and this condition is related to the functional capacities of the peripheral immunocompetent cells rather than to thymic defects [17–22].

Accordingly, in an attempt to clarify the immune condition of EphB-deficient mice that generate their T-cell repertoire in a thymus with profoundly altered thymocyte-TEC interactions, we examine changes in the proportions of intrathymic B lymphocytes, DC subsets and mFBs, as well as the possible development of autoimmune processes in EphB2 and EphB3 knock out (KO) mice, evaluated by the appearance of lymphoid infiltrates in different organs, the presence of circulating autoantibodies, and the capacity to develop chicken type II collagen-induced arthritis (CIA).

## Methods

### Animals

Two-month-old WT, EphB2^−/−^, and EphB3^−/−^ male mice (*Mus musculus*) were used in the current study. Mutant mice were kindly provided by Dr. Mark Henkemeyer (University of Texas, Southwestern Medical Center at Dallas) generated in a CD1 outbred background. All animals were maintained under sterile conditions in the animal housing facilities of the Complutense University of Madrid (UCM), and the experimental procedures developed in accordance with the regulations of the Ethic Committee for Animal Research of UCM and the Regional Government of Madrid.

### Cell suspensions

Thymuses and inguinal lymph nodes (ILNs) were collected, cleaned of surrounding connective and adipose tissue and mechanically disaggregated using a homogenizer with RPMI 1640 medium at 2% fetal bovine serum (FBS). Cell suspensions were filtered to remove cellular debris.

For both thymic dendritic cell and medullary fibroblast analysis, previous cell enrichment was carried out. For this purpose, clean thymic lobes were cut with fetal scissors and transferred into a Falcon tube containing cold 1 × phosphate buffered saline (PBS). They were gently pipetted to promote release of the contained thymocytes, and the final fragments were enzymatically disrupted using Liberase TM (Roche) at 1 U/mL together with DNAse I at 0.1 mg/mL (Roche) for 20 min (min.) at 37 °C. Fragments were completely disaggregated mechanically, and the cell suspension was filtered and washed in FACS buffer (PBS with 2% FBS and 10 mM EDTA), centrifuging at 1500 r.p.m. for 5 min. at 4 °C. Finally, the pellet was resuspended in FACS buffer.

In all cases, cell counting was performed in a Neubauer-type hemocytometer discarding the dead cells.

### Flow cytometry analysis

Cell suspensions (0.5–1 × 10^6^ cells) were stained with specific antibodies (Online Resource 1) for 15 min. at 4 °C in darkness. After incubation, samples were washed with PBS and centrifuged at 1500 r.p.m. for 5 min. at 4 °C. For detection of intracellular markers (FoxP3, IFNɣ, IL2, IL4, IL17), after superficial antigen staining, cell suspensions were fixed and permeabilized overnight at 4 °C with CytoFix/CytoPerm solution (BD Biosciences or eBiosciences). Then, samples were washed with PermWash solution (BD Biosciences or eBiosciences) at 1500 r.p.m. for 5 min. at 4 °C. Pellets resuspended in PermWash containing specific antibodies for these molecules were incubated for 30 min. at 4 °C. Finally, samples were washed twice in PermWash, resuspended in PBS, and analyzed in a flow cytometer FACS Calibur or FACS Aria III (BD Biosciences) from the Cytometry and Fluorescence Microscopy Center of the UCM. Dead cells were excluded from the analysis according to size (FSC) and complexity (SSC) parameters. All data obtained were analyzed with the FCS Express III (De Novo) software.

Previously to immunodetection of cytokine markers (IFNɣ, IL2, IL4, IL17), 1 × 10^6^ ILN cells were cultured for 5 h at 37 °C and 5% CO_2_ in 48-multi-well-plate and stimulated in vitro using complete culture medium (RPMI 1640, 10% FBS, 1% Glutamine, 1% Penicillin/Streptomycin, 1% Sodium pyruvate, 1 × β-mercaptoethanol) supplemented with 5 ng/mL phorbol myristate acetate (PMA) and 495 ng/mL Ionomycin (Sigma-Aldrich), adding after the first hour GolgiStop™ and GolgiPlug™ (BD Biosciences). After this time, the wells were washed, and transferred to FACS tubes for subsequent analysis, as above indicated, by flow cytometry.

### Collagen-induced arthritis (CIA) assay

For the induction of arthritis in both outbred CD1 WT and EphB-deficient mice, two subcutaneous tail injections were performed 21 days apart, using 0.1 mL of a chicken type II collagen (Chondrex) emulsified with complete Freund's adjuvant (CFA) (Chondrex). CFA (2 mg/ml) and collagen (2 mg/ml) were mixed thoroughly at a 1:1 ratio and emulsified using a syringe to obtain a dense cream consistency. Animals were monitored to identify signs of arthritis (joint or digit inflammation) once a week from the first immunization and twice a week from the second immunization. Animals were sacrificed at day 34 or 48 after first immunization.

### Histological analysis

To determine the presence of lymphoid infiltrates in testis, adipose tissue, liver and submandibular salivary gland from either WT or EphB-deficient mice, samples were isolated, cleaned and fixed in paraformaldehyde solution 4% in water for 12 h, and washed in 70° ethanol. For histological analysis of animals used in CIA model, paws were dissected, isolated of surrounding skin and muscle tissue and fixed in formaldehyde solution 4% in water overnight. Then, samples were decalcified with Osteosoft^®^ solution (Merck) for 1 week and finally washed in PBS.

In both cases, samples were included in paraffin and sectioned in a microtome to obtain sections 10 µm thick that were stained with hematoxylin for 10 min., then washed in water and stained with eosin for 5 min. Finally, sections were washed, dehydrated in increasing alcohol gradients, and preserved using DPX mountant. Sections were analyzed using a Zeiss Axioskop microscope with DP controller software from the Cell Biology Department of UCM.

### Detection of serum autoantibodies using histological sections

The presence of autoantibodies (IgG) was detected in serum samples obtained from either WT or EphB-deficient mice. For this proposal, WT submandibular salivary glands were isolated, embedded in Tissue-Tek OCT compound (Sakura), and snap frozen using liquid nitrogen. 10 μm-thick tissue sections obtained in a cryostat were fixed in acetone for 10 min. at room temperature (RT) and air dried. WT salivary sections were incubated with WT or EphB-mutant sera (1/50) for 45 min. at RT. After washing 3 times in cold PBS for 5 min., the presence of serum IgG on the tissue was detected using a Rabbit (Fab)_2_ anti-mouse IgG^AlexaFluor488^ (Jackson ImmunoResearch) for 45 min. Then, sections were washed three times in cold PBS for 5 min. and mounted with SlowFade™ Diamond Antifade Mountant (ThermoFisher Scientific). Images were acquired with a Zeiss Axioplan II microscope from the Cytometry and Fluorescence Microscopy Center of the UCM.

### In vitro T-cell responses

2 × 10^5^ ILN total cells, obtained as described above, were resuspended in complete culture media, plated on U-bottom 96-well plates (200 µL/well), supplemented with Dynabeads™ Mouse T-Activator CD3/CD28 (ThermoFisher Scientific), according to the manufacturer’s instructions, and cultured in 5% CO_2_ for 72 h at 37 °C.

CD3/CD28-mediated T-cell activation was determined using Cell Proliferation Reagent WST-1 (Merck) that indicates the rate of cell proliferation. After 72 h of cell culture, 20 µL of WST-1 was added to each well and incubated for 4 h. After this period, the formazan dye formed was quantified by optical density measured at 450 nm with a Biochrom^®^ Asys UVM340 microplate reader that directly correlates to the number of viable cells.

### Detection of mouse IgG anti-chicken type II Collagen

The presence of anti-type II collagen antibodies in the serum of WT and EphB-mutant mice was evidenced by ELISA using Mouse Anti-chicken type II Collagen IgG Antibody ELISA kits with TMB (Chondrex). According to manufacturer’s instructions, serum samples were diluted, and optical densities were read in a Biochrom^®^ Asys UVM340 microplate reader at 450 nm. The determination of antibody titers (U/mL) was calculated using regression analysis and the sample dilution.

### In vitro cell migration assay

WT and EphB-mutant ILN obtained one day after the second immunization with chicken type II collagen were processed to obtain a total cell suspension. Then, T-cell isolation was performed using Dynabeads^®^ Untouched Mouse T cells kit (ThermoFisher Scientific) according to the manufacturer’s instructions. For in vitro migration assays, 0.5 × 10^6^ T cells were diluted in 100 μl of RPMI BSA 1% and seeded on 24-well transwell plates containing nitrocellulose inserts with 5 μm pore size (Corning Costar). The lower compartment was filled with 600 μl of RPMI BSA 1% supplemented with CXCL12 chemokine (100 ng/ml) (Biolegend). Cells were cultured for 5 h at 37 °C and 5% CO_2_. Then, the lower medium containing migrating cells was collected, centrifuged for 5 min. at 1500 r.p.m. and the pellet stained with specific antibodies. All cells were resuspended in the same volume and acquired by flow cytometer using the same conditions of time and flux speed. The percentage of migration was calculated by dividing the number of recovered cells by the initial number of seeded cells in the inserts × 100.

### Statistical analysis

The obtained results were expressed as the mean ± standard deviation (SD). Firstly, the values were subjected to a normality test (D'Agostino-Person test) and homoscedasticity test (Brown–Forsythe test) by using GraphPad Prism 8 (San Diego, California). When data were normal and homoscedastic, the one-way ANOVA test was applied; if they were normal but heteroscedastic the ANOVA Brown–Forsythe and Welch test was used, and for those that were not normal, a non-parametric Kruskal–Wallis test was applied, followed by the post-hoc Sidak, Tukey, Tamhane T2 or Dunn's test, respectively. The statistical significance (*p*-value) was indicated as: *p < 0.05; **p < 0.01; ***p < 0.001; ****p < 0.0001.

## Results

### Changes in the proportions of thymic non-epithelial antigen-presenting cells involved in the negative selection of thymocytes

**Fig. 1:**
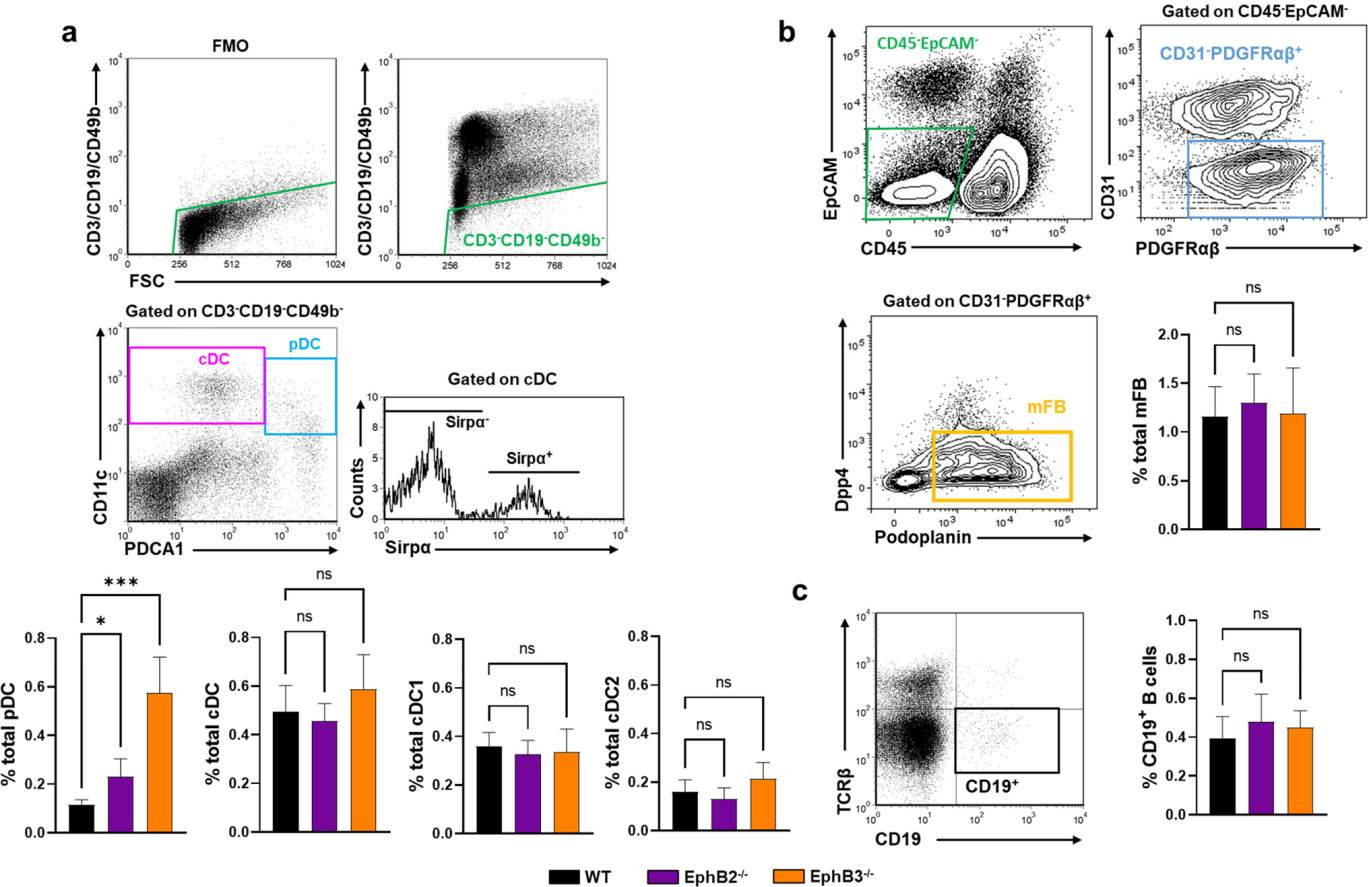
Proportions of thymic non-epithelial antigen presenting cells in both WT and EphB-deficient mice. **a** Dot plots show gating sequence for thymic dendritic cell subset analysis. CD3^−^CD19^−^CD49b^−^ cells were considered on the basis of fluorescence minus one control (FMO). Plasmacytoid dendritic cells (pDC) and conventional dendritic cells (cDC) were defined according to CD11c and PDCA1 marker expression and gated on CD3^−^CD19^−^CD49b^−^ cells. cDC subsets (cDC1 and cDC2 cells) were identified according to Sirpα expression gated on the cDC subset. The figures show the percentages of total pDCs (CD3^−^CD19^−^CD49b^−^CD11c^+^PDCA1^+^ cells), total cDC (CD3^−^CD19^−^CD49b^−^CD11c^+^PDCA1^−^ cells) and the proportions of cDC subsets, Sirpα^−^ cells (cDC1) and Sirpα^+^ cells (cDC2). **b** Dot plots indicate the gating protocol for medullary fibroblast (mFBs) analysis. The graph represents the proportions of total CD45^−^EpCAM^−^CD31^−^PDGFRαβ^+^Dpp4^−^Podoplanin^+^ mFBs. **c** Representative dot plot of thymic B cells (CD19^+^TCRβ^−^) and their total proportions. At least 4 (WT), 3 (EphB2^−/−^) and 3 (EphB3^−/−^) mice were analyzed. Data are presented as mean ± standard deviation (SD) and analyzed using the Kruskal–Wallis nonparametric test with Dunn’s post hoc test (**b**, **c**), Brown–Forsythe and Welch ANOVA test with Tamhane’s T2 post hoc test (**a**; total pDC) and one-way ANOVA test with Tukey’s post hoc test (**a**; total cDC, total cDC1, total cDC2). *p < 0.05; and ***p < 0.001; ns: not significant

Apart from the medullary TECs, non-epithelial APCs including plasmacytoid DCs (pDCs), Type 1 and Type 2 conventional DCs (cDCs), medullary fibroblasts (mFBs) and B (CD19^+^) cells are major protagonists in the establishment of central immune tolerance. Therefore, we evaluated, by flow cytometry (Fig. 1), possible changes in their proportions in the thymuses of EphB2- or EphB3-deficient mice, as compared to control, WT, ones, rather than absolute values because EphB2- and EphB3-deficient mice show a profound thymic hypocellularity [4, 5] that masks the possible differences.

Within the CD3^−^CD19^−^CD49b^−^ cell subset, the expression of CD11c and PDCA1 cell markers allowed to distinguish CD11c^+^PDCA1^−^ cDCs and CD11c^+^PDCA1^+^ pDCs (Fig. 1a). In addition, cDCs comprise two cell subpopulations: CD11c^+^PDCA1^−^Sirpα^−^ (cDC1) cells and CD11c^+^PDCA1^−^Sirpα^+^ (cDC2) cells (Dot plot gating strategy Fig. 1a). Variations from control WT values appeared in the EphB-deficient thymuses in which the proportions of pDCs increased, but not those of cDC subsets, nor the percentage of total thymic cDCs (Fig. 1a). In addition, we evaluated the total proportions of mFBs and B cells (Dot plot gating strategy Fig. 1b, c, respectively) there were no changes in the proportions of total CD45^−^EpCAM^−^CD31^−^PDGFRαβ^+^Dpp4^−^Podoplanin^+^ mFBs (Fig. 1b), or in those of CD19^+^ B lymphocytes in mutant thymuses (Fig. 1c).

### The organs of EphB-deficient mice did not exhibit either lymphoid infiltrates or circulating autoantibodies

**Fig. 2:**
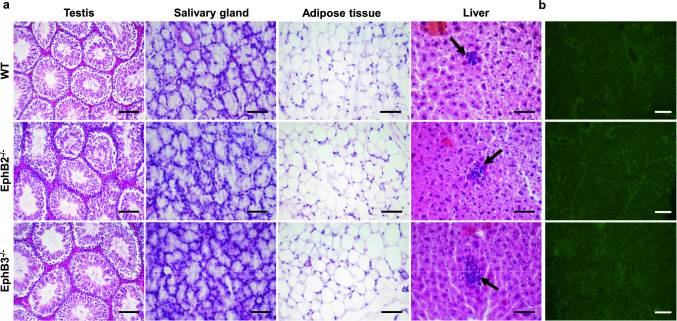
Histological sections of testis, salivary gland, adipose tissue, and liver from WT, EphB2^−/−^ and EphB3^−/−^ mice stained with hematoxylin–eosin (**a**). Note the presence of small lymphoid infiltrates only in WT and EphB-mutant livers (arrows). Scale: 100 µm (testis) and 50 µm (salivary gland, adipose tissue, and liver). **b** Immunofluorescence detection of IgG autoantibodies on WT submandibular salivary gland sections after incubation with serum obtained from WT, EphB2^−/−^ and EphB3^−/−^ mice. Note the absence of fluorescence. Sections are representative from 3 independent WT, EphB2^−/−^ and EphB3^−/−^ mice. Scale: 100 µm

In an attempt to evaluate possible functional alterations in the immune education undergone by thymocytes generated in the EphB-deficient thymuses, we analysed histologically the presence of lymphocyte infiltrates in testis, salivary glands, adipose tissue and liver in both mutant and WT mice (Fig. 2a). No lymphoid accumulations occurred in these organs; only the appearance of some small hepatic infiltrates, mainly close to the blood vessels, that occurred in both EphB KO and control WT mice (Fig. 2a).

In order to confirm these results, we analyzed the possible presence of autoantibodies in the serum of mutant mice capable of recognizing specific tissue antigens. For this propose, histological sections of salivary glands of WT mice were incubated with sera isolated from either WT or EphB-deficient mice and reactivity was evidenced with an anti-IgG antibody. No fluorescence was observed in the presence of mutant sera (Fig. 2b), indicating the lack of autoantibodies.

### Response of EphB-deficient mice to the administration of chicken type II collagen

**Fig. 3:**
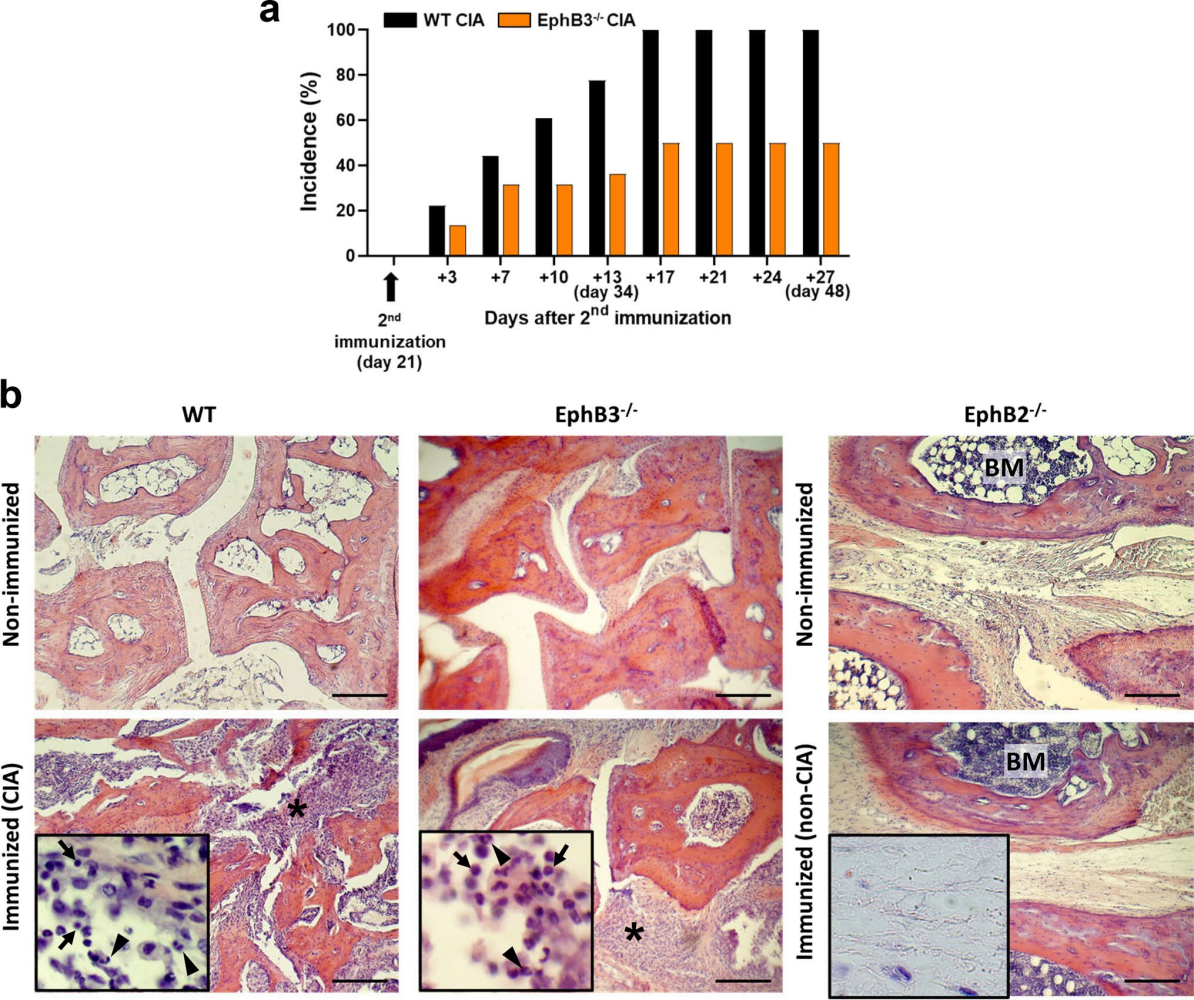
Incidence of arthritis in both WT and EphB3^−/−^ mice after second immunization with chicken type II collagen and CFA (**a**). Note the lower susceptibility of EphB3^−/−^ mice to suffer CIA compared to WT animals. EphB2^−/−^ mice did not developed arthritis and they are not included in the graph. 18 WT, 14 EphB2^−/−^ and 22 EphB3^−/−^ immunized mice were studied. **b** Histological analysis of paw sections stained with hematoxylin–eosin from immunized animals with chicken type II collagen 34 days post immunization. Note the presence of arthritic lesions containing cell infiltrates (*) in both immunized WT and EphB3^−/−^ mice (CIA) respect to non-immunized but their absence in EphB2^−/−^ mice (non-CIA). Inserts show presence of mononuclear cells (arrows) and neutrophils (arrowheads) in WT and EphB3^−/−^ immunized (CIA) mice and their absence in EphB2^−/−^ immunized (non-CIA) mice. Images are representative of 3 independently analyzed animals of each condition. Scale bar: 200 µm. BM: bone marrow

Outbred CD1 WT and mutant mice were subcutaneously immunized twice 21 days apart in the tail with 0.1 ml of chicken type II collagen (2 mg/ml) in CFA (1:1). As shown in Fig. 3a, 34 days after the first immunization 77% WT animals showed arthritic lesions consisting of connective tissue (Fig. 3b) containing cell infiltrates (Fig. 3b, insert). A similar condition occurred in EphB3^−/−^ mice, but only 36% of mutants developed arthritis (Fig. 3a, b, insert). On the other hand, EphB2^−/−^ mice did not exhibit lesions, showing a loose connective tissue (Fig. 3b) devoid of cell infiltrates (Fig. 3b, insert). When the animals were tested at different stages from day 34 to day 48 after primary immunization, all stimulated WT mice (100%) developed arthritis. Notably, from day 17 after the second immunization, when all the WT mice had fallen ill, only half the EphB3-deficient animals had become sick, and this number did not increase in the following days (Fig. 3a). Therefore, the onset of lesions was quite similar in WT and EphB3^−/−^ mice but the number of sick mice within the WT mouse group quickly increased.

**Fig. 4:**
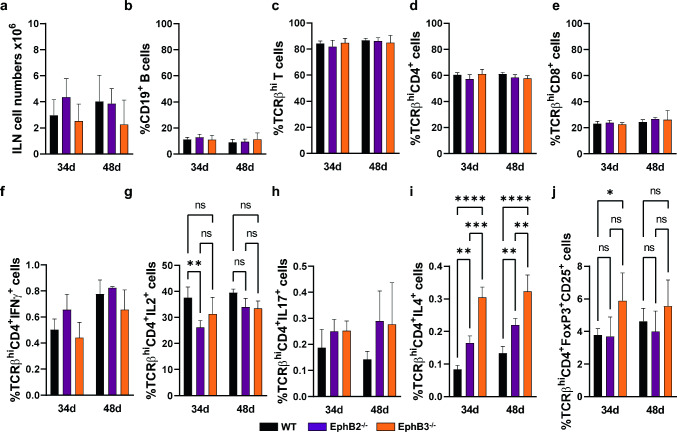
ILN cellular content and proportions of different cell subsets in control non-immunized WT, EphB2^−/−^ and EphB3^−/−^ mice on days 34 and 48. **a** Total ILN cellularity. **b** Percentage of CD19^+^ B cells. **c** Percentage of TCRβ^hi^ T cells, total TCRβ^hi^CD4^+^ cells (**d**) and TCRβ^hi^CD8^+^ cells (**e**). Proportions of Th1 cells (TCRβ^hi^CD4^+^IFNɣ^+^ cells (**f**) and TCRβ^hi^CD4^+^IL2^+^ cells (**g**)), **h** Th17 cells (TCRβ^hi^CD4^+^IL17^+^ cells) and **i** Th2 cells (TCRβ^hi^CD4^+^IL4^+^ cells) after in vitro stimulation with PMA and ionomycin. **j** Percentage of CD4^+^ T regulatory cells (TCRβ^hi^CD4^+^FoxP3^+^CD25^+^ cells). At least 3 independent animals were analyzed from each condition (WT, EphB2^−/−^ and EphB3^−/−^) and time (34 and 48 days). Data are presented as mean ± standard deviation (SD) and analyzed using one-way ANOVA test with Tukey’s post hoc test. *p < 0.05; **p < 0.01; ***p < 0.001 and ****p < 0.0001; ns: not significant

**Fig. 5:**
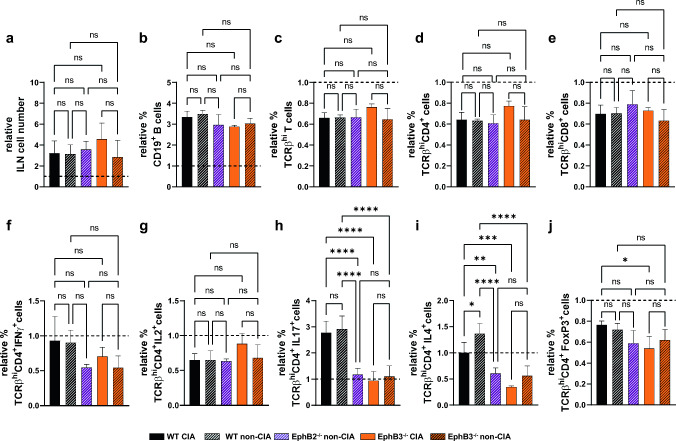
Changes in the cellularity and the relative proportions of different T cell subsets from draining inguinal lymph nodes (ILN) in both immunized WT and EphB-deficient mice with chicken type II collagen at day 34. WT and EphB3^−/−^ sick animals (CIA) and WT, EphB2^−/−^ and EphB3^−/−^ healthy (non-CIA) mice are analyzed. All shown data are related to the values obtained in immunized mice respect to their non-immunized controls. **a** Relative ILN cell numbers. **b** Relative proportions of CD19^+^ B cells. Relative percentage of TCRβ^hi^ T cells (**c**), total TCRβ^hi^CD4^+^ cells (**d**), and TCRβ^hi^CD8^+^ cells (**e**). Relative percentage of Th1 cells ((**f**) TCRβ^hi^CD4^+^IFNɣ^+^ cells and TCRβ^hi^CD4^+^IL2^+^ cells (**g**)), Th17 cells (TCRβ^hi^CD4^+^IL17^+^ cells) (**h**) and Th2 cells (**i**) (TCRβ^hi^CD4^+^IL4^+^ cells) after in vitro stimulation with PMA and ionomycin. **j** Relative proportions of CD4^+^ T regulatory cells (TCRβ^hi^CD4^+^FoxP3^+^CD25^+^ cells). At least 3 (WT CIA), 3 (WT non-CIA), 4 (EphB2^−/−^ non-CIA), 3 (EphB3^−/−^) and 5 (EphB3^−/−^ non-CIA) animals were analyzed. Data are presented as mean ± standard deviation (SD) and analyzed using Brown–Forsythe and Welch ANOVA test with Tamhane’s T2 post hoc test (**b**) one-way ANOVA test with Sidak’s post hoc test (**a**, **c**–**j**). *p < 0.05; **p < 0.01; ***p < 0.001 and ****p < 0.0001; ns: not significant

**Fig. 6:**
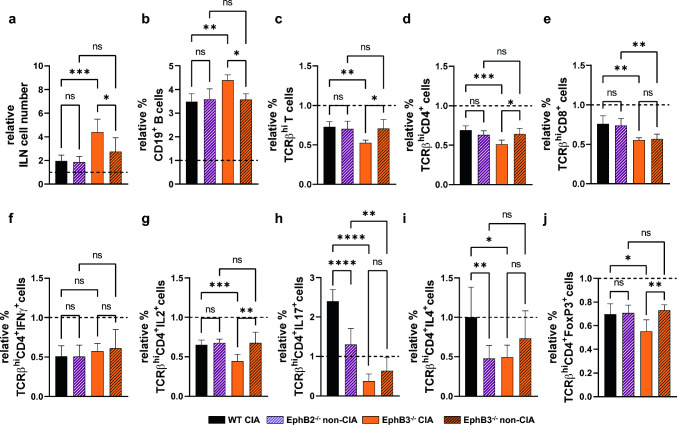
Effect of chicken type II collagen immunization on the cell content and the relative proportions of different T cell subsets from draining inguinal lymph nodes (ILN) in both WT and EphB-deficient mice at day 48 after first immunization. WT and EphB3^−/−^ sick animals (CIA) and EphB2^−/−^ and EphB3^−/−^ healthy (non-CIA) mice are studied. All shown data are related to the values obtained in immunized mice relative to their non-immunized control. **a** Relative ILN cell numbers. **b** Relative proportions of CD19^+^ B cells, TCRβ^hi^ T cells (**c**), TCRβ^hi^CD4^+^ cells (**d**) and TCRβ^hi^CD8^+^ cells (**e**). Relative percentage of Th1 cells ((**f**) (TCRβ^hi^CD4^+^IFNɣ^+^ cells) and TCRβ^hi^CD4^+^IL2^+^ cells (**g**)), Th17 cells (TCRβ^hi^CD4^+^IL17^+^) (**h**) and Th2 cells (**i**) (TCRβ^hi^CD4^+^IL4^+^ cells) after in vitro stimulation with PMA and ionomycin. **j** Relative proportions of CD4^+^ T regulatory cells (TCRβ^hi^CD4^+^FoxP3^+^CD25^+^ cells). At least 8 (WT CIA), 6 (EphB2^−/−^ non-CIA), 3 (EphB3^−/−^) and 3 (EphB3^−/−^ non-CIA) animals were analyzed. Data are presented as mean ± standard deviation (SD) and analyzed using one-way ANOVA test with Sidak’s post hoc test. *p < 0.05; **p < 0.01; ***p < 0.001 and ****p < 0.0001; ns: not significant

We then evaluated possible changes in lymphoid cell populations known to be involved in autoimmunity, including distinct T-cell subsets and B lymphocytes [23–25]. For this proposal, we analyzed by flow cytometry the T and B cell subsets present in the draining inguinal lymph nodes (ILN) in order to establish a profile of the lymphoid cells involved in the appearance of lesions (Fig. 4). Because we did not observe significant differences in the ILN cellularity of WT and EphB-mutant mice (Fig. 4a), we analyzed the total proportions of different cell subsets (see Dot plot gating strategy in Online Resource 2) rather than their absolute numbers (Fig. 4). Although variations were not pronounced, there were some differences in the proportions of certain cell subsets between non-immunized WT and mutant mice, presumably reflecting the outbred background of the used CD1 mouse strain [26]. There appeared to be an initial seeking of less reactive mutant T cells than of WT mice. Thus, on day 34, there were higher IL2-producing cells in non-immunized WT mice than in EphB2^−/−^ ones (Fig. 4g), but the proportions of Treg cells were higher in EphB3-deficient mice (Fig. 4j) and in all stages analyzed values of IL4-producing cells were higher in both EphB2 and EphB3 mutants than in WT mice (Fig. 4i). To reduce this initial variability between non-immunized WT and EphB-mutant mice, all data of immunized mice were randomized with respect to those of the non-immunized animals (Figs. 5, 6). Accordingly, all results obtained in our study were compared with the respective control values of non-immunized WT and mutant mice.

We established the following experimental groups: WT mice with arthritis (WT CIA) or without it (WT non-CIA), EphB2 mutant mice that did not suffer the disease (EphB2^−/−^ non-CIA) and EphB3^−/−^ mice that developed the disease (EphB3^−/−^ CIA) or not (EphB3^−/−^ non-CIA), and the following comparative analyses were carried out:

1. Phenotypical profiles of WT mice developing arthritis (WT CIA) versus those of healthy ones (WT non-CIA).
2. Presumptive changes that affected the sick EphB3^−/−^ mice (EphB3^−/−^ CIA) versus those that did not develop arthritis (EphB3^−/−^ non-CIA).
3. Condition of EphB2^−/−^ mice that did not suffer CIA (EphB2^−/−^ non-CIA). Results will be compared with those of healthy WT (WT non-CIA) and EphB3^−/−^ mice (EphB3^−/−^ non-CIA), as well as with WT mice developing arthritis (WT CIA).

### Changes in the lymphoid cell populations of WT CIA mice

Thirty four days after immunization, WT CIA animals experienced increased numbers of total cells (Fig. 5a) and relative proportions of B lymphocytes in the draining ILN (Fig. 5b). They also presented decreased proportions of total mature TCRβ^hi^ T lymphocytes (Fig. 5c) and of subsets defined by the expression of CD4 (Fig. 5d) and CD8 cell markers (Fig. 5e) compared to control, non-immunized WT mice. However, there were no significant differences between the values observed in mice developing arthritis and in healthy ones. Likewise, there were no variations between the relative proportions of IFNγ- (Fig. 5f) and IL2-producing cells (Fig. 5g), exhibiting values under the levels of control, non-immunized mice in both WT CIA and WT non-CIA mice. The relative proportions of IL17^+^ cells increased almost 3 times compared to control values, but also increased in the WT non-CIA mice (Fig. 5h). However, the relative proportions of IL4-producing cells were significantly higher in WT non-CIA than in WT mice developing arthritis (Fig. 5i), that would downregulate their immunoreactivity. Remarkably, the proportions of Treg cells were under control levels of non-immunized mice in both groups of injected mice (Fig. 5j).

We could not analyze comparatively the condition of sick WT (WT CIA) mice and WT non-CIA mice on day 48, because, as indicated, all immunized WT mice had developed arthritis at that time stage, Therefore, we compared the phenotypic profile of WT CIA mice with that of control, non-immunized WT ones and then, with that of the arthritis suffering EphB3-mutant mice.

In general, the results suggested that, as previously reported, the incidence of disease was significantly higher in WT CIA mice than in EphB3^−/−^ CIA ones (Fig. 3a). In this latter experimental group, from day 34 onward the number of arthritic mice remained stable, although half of the immunized EphB3-mutant mice were still healthy (Fig. 3a), suggesting a lower immunocompetence of EphB3-deficient mice for inducing arthritic lesions. On day 48, the cellularity of WT CIA mice (Fig. 6a) reduced compared to the values found on day 34 (Fig. 5a), presumably reflecting that reactive lymphoid cell had reached the paws and/or showed lower anti-collagen II reactivity, since all immunized WT mice had already fallen ill (Fig. 3a). On the contrary, the relative numbers of EphB3^−/−^ ILN cells did not decrease (Fig. 6a). Their relative proportions of total TCRβ^hi^ T cells, TCRβ^hi^CD4^+^ cells, and TCRβ^hi^CD8^+^ cells (Fig. 6c–e) were significantly lower than those of WT CIA mice, but the relative frequency of EphB3^−/−^ CIA B lymphocytes increased significantly (Fig. 6b). To support this, WT mice contained higher proportions of IL2- (Fig. 6g) and IL17-producing cells (Fig. 6h), but also IL4-producing cells (Fig. 6i) and Treg cells (Fig. 6j) than EphB3^−/−^ CIA mice, which would regulate the self-reactivity of these proinflammatory T cell subsets.

### Comparative analysis of the lymph node lymphoid cell subsets of EphB3^−/−^ CIA mice and EphB3^−/−^ non-CIA mice

On day 34, there were no significant variations in the proportions of most of the immune cell subsets analyzed between the EphB3-deficient mice which developed arthritis, and those that remained healthy (Fig. 5). On day 48, the proportions of B lymphocytes increased (Fig. 6b), whereas those of total TCRβ^hi^ T cells (Fig. 6c) and TCRβ^hi^CD4^+^ cells (Fig. 6d) decreased relative to the values observed in EphB3^−/−^ non-CIA animals. However, the relative numbers of total ILN cells increased (Fig. 6a) suggesting that T-cell activation predominated in cell migration to the joints in this group of animals. In addition, the proportion of IL2-producing cells were significantly higher in the EphB3 mutants that did not develop the disease (Fig. 6g), but these animals also showed higher proportions of Treg cells than the sick ones (Fig. 6j).

### The immune profile of EphB2^−/−^ mice that did not develop arthritis (EphB2^−/−^ non-CIA) as compared with both WT and EphB3^−/−^ healthy mice and WT CIA animals

Remarkably, EphB2^−/−^ non-CIA ILN contained quite similar proportions of distinct immune cell subsets and the changes observed were quite similar on day 34 and day 48 (Figs. 5, 6). At day 34, the proportions of IL4-producing cells were significantly lower than those found in both WT non-CIA and WT CIA mice (Fig. 5i), but similar to the percentage of EphB3^−/−^ non-CIA (Fig. 5i). However, on day 48, this last value was also higher in WT-CIA ILN than that of EphB2^−/−^ non-CIA (Fig. 6i). Furthermore, on day 34, the proportions of Th17 cells were significantly lower in EphB2^−/−^ non-CIA than both WT CIA and WT non-CIA mice (Fig. 5h), but higher than those of EphB3^−/−^ non-CIA mice on day 48 (Fig. 6h). This paucity of phenotypical changes suggests that perhaps the absence of sick EphB2^−/−^ animals could be due to an altered functional capacity, such as a lack of immune reactivity against the chicken type II collagen and/or to migratory problems that impede this getting to the joints thus favoring arthritic lesions, as reported for other Eph and ephrins [22]. Accordingly, we have studied these two functions in the above-mentioned experimental groups.

**Fig. 7:**
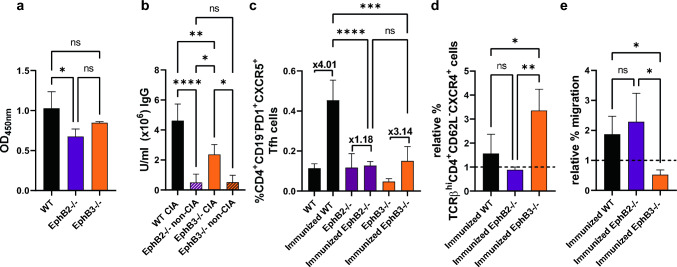
WST-1 assay to test the proliferative capacities of ILN T-cells 3 days after in vitro stimulation with Dynabeads™ Mouse T-Activator CD3/CD28 (**a**). OD: Optical density. Analyzed data n = 3. **b** Serum levels (U/mLx10^6^) of anti-chicken type II collagen IgG antibodies in immunized WT and EphB-mutant mice analyzed at day 48 post first immunization and measured by ELISA. WT CIA (n = 9), EphB2^−/−^ non-CIA (n = 5), EphB3^−/−^ CIA (n = 3) and EphB3^−/−^ non-CIA (n = 5) samples were analyzed. **c** Frequencies of T follicular helper cells (Tfh) (CD4^+^CD19^−^PD1^+^CXCR5^+^ cells) in non-immunized and immunized WT and EphB-deficient mice. Notice the increased fold change between non-immunized animals and their immunized counterparts in the case of WT and EphB3^−/−^ mice but not in EphB2^−/−^ animals. Analyzed data n = 3. **d** Relative proportions of TCRβ^hi^CD4^+^CD62L^−^CXCR4^+^ cells and **e** their relative percentage of migration in immunized both WT and EphB-mutant mice respect their control, non-immunized counterparts. At least 5 (immunized WT), 3 (immunized EphB2^−/−^) and 3 (immunized EphB3^−/−^) animals were analyzed. Data are presented as mean ± standard deviation (SD) and analyzed using one-way ANOVA test with Sidak (**b**, **c**) or Tukey’s (**a**, **d**, **e**) post hoc test. *p < 0.05; **p < 0.01; ***p < 0.001 and ****p < 0.0001; ns: not significant

Firstly, we used WST-1 reagent to evaluate the proliferative capacities of T lymphocytes derived from either WT or EphB-deficient (both EphB2^−/−^ and EphB3^−/−^ cells) lymph nodes stimulated in vitro for 72 h with specific anti-CD3 and anti-CD28 antibodies. As shown in Fig. 7a, no significant differences occurred when EphB3^−/−^ lymphocytes were tested versus WT ones, but those derived from EphB2-deficient lymph nodes proliferated significantly less than the stimulated WT T cells.

Because autoantibodies contribute significantly to the pathogenesis of rheumatoid arthritis (RA) [27, 28], at 48 days post-immunization we analyzed, by ELISA, the presence of specific anti-type II collagen IgG antibodies in the serum of WT and mutant mice. Supporting a certain reduction in the immune reactivity of EphB-deficient mice, the serum antibody titers were statistically higher in WT mice that developed arthritis than in EphB3^−/−^ CIA mice, and particularly, than in mutant mice unable to induce arthritis, such as both EphB2^−/−^ non-CIA and EphB3^−/−^ non-CIA mice (Fig. 7b). In agreement with these results, the proportions of CD4^+^CD19^−^PD1^+^CXCR5^+^ T follicular helper cells (Tfh), which regulate the development of germinal centers and the production of plasma cells and memory B cells [29], were significantly higher in immunized WT than in both immunized EphB2- and EphB3-deficient mice (Fig. 7c). Remarkably, whereas the values of Tfh cells in immunized WT mice were fourfold higher than in non-stimulated ones (Fig. 7c) and threefold higher in EphB3^−/−^ animals than in their non-immunized counterparts (Fig. 7c), EphB2-deficient mice, which were unable to induce arthritic lesions, did not show changes in the proportions of Tfh cells present in the ILNs after immunization (Fig. 7c).

Finally, we studied the frequency of TCRβ^hi^CD4^+^CD62L^−^ cells that express CXCR4, the chemokine receptor of CXCL12 involved in the migration of immunocompetent cells to arthritic lesions [30] derived from the immunized ILN, relative to the values of their respective control, non-stimulated animals. In these conditions, the relative proportions of TCRαβ^hi^CD4^+^CD62L^−^CXCR4^+^ cells were significantly higher in immunized EphB3^−/−^ mice than in both the immunized EphB2^−/−^ and control WT mice (Fig. 7d). Remarkably, the relative migration of these cells in transwell assays compared with those isolated from their respective non-immunized controls were significantly lower in EphB3-deficient mice (Fig. 7e). Taking all these results together, EphB3 mutants appear to present important migration problems, whereas EphB2-deficient mice exhibit difficulties to be adequately activated after type II collagen administration. Both findings would explain the observed deficits in the response to type II collagen immunization and the partial or total absence of arthritis in these mutants, respectively.

## Discussion

Our current study is an attempt to clarify the immune condition of EphB2- and EphB3-deficient mice that in previous studies have been demonstrated to exhibit profound alterations in the thymic epithelial network [31–34], but few phenotypical variations in the proportions of different thymic T cell subsets, including those involved in positive (TCRαβ^hi^CD4^+^CD8^+^CD69^+^ cells, TCRαβ^hi^CD4^+^CD8^−^CD69^+^ cells) and negative selection (Caspase3^+^CD5^+^CD69^+^CD4^+^CD8^+^ cells, Caspase3^+^CD5^+^CD69^+^CD4^+^CD8^−^ cells), as well as in the frequency of Treg cells (TCRαβ^hi^FoxP3^+^ cells, TCRαβ^hi^FoxP3^+^CD4^+^ cells) [3–5].

Because the lack of alterations in the phenotypical differentiation of thymocytes, or even in the peripheral T lymphocytes, does not invariably mean an accurate immune reactivity, we have designed various experimental approaches to determine whether altered thymocyte-TEC interactions would affect the immune capabilities of our mutants [5] or, as previously suggested, a low number of thymic cell-to-cell interactions would be sufficient to ensure a normal functioning of the immune system [35].

Other authors had formulated a similar hypothesis [6–10], although it was also remarked that the lack of thymocyte-TEC interactions might be supplied by other APCs, mainly DCs, also involved in intrathymic immune education [11]. Accordingly, we first evaluated possible changes in the presence of APCs, including various DC subsets, B lymphocytes and mFBs, known for their involvement in intrathymic T cell selection [13, 15, 36]. However, only the proportions of pDCs increase in the thymuses of EphB2- and EphB3-deficient mice. Moreover, the immune functions of pDCs are controversial, particularly their capacities as APC [37] and their origin [38].

Although we had observed that EphB-deficient mice appeared healthy, without signs of autoimmunity [12], we analyzed the presence of lymphoid cell infiltrates in several organs, the occurrence of serum autoantibodies and the development of chicken type II-collagen mediated arthritis. No lymphoid cells appear in testes, salivary glands or adipose tissue and some small lymphoid accumulations found in some liver sections of mutant mice also occur in WT livers, as previously reported [39]. On the other hand, no reactivity to self-tissue antigens was observed when histological sections of WT salivary glands were incubated with serum from either WT or EphB-deficient mice.

In order to confirm this apparent lack of autoimmune reactivity in the EphB mutant mice, we use an experimental model for inducing arthritis by subcutaneous injection of chicken type II collagen in both WT and EphB-deficient mice. RA is a polygenic autoimmune disease that mainly affects the joints. Its pathogenesis is not fully known, involving among other factors: Toll-like receptors (TLRs), autoantibodies and some inflammatory cytokines and their producing cells, such as Th17 cells [40–42], although there is little evidence to relate T-cell phenotyping and disease progression [24].

As reported by other authors in similar models in which arthritis or experimental autoimmune encephalomyelitis (EAE) is induced in mice deficient in EphA4 [21], or in ephrin-B1, ephrin-B2 or ephrin-B1/B2, main ligands of EphB2 and EphB3 [19, 22, 43], the immune response was remarkably low in both EphB2- and EphB3-deficient mice resulting in a low incidence of disease, and was totally null in EphB2^−/−^ mice. Ameliorated development of EAE [19] and CIA [22] have been reported in ephrin-B1 and ephrin-B2 double KO, two of the main ligands of EphB2 and EphB3 analyzed in the current study, suggesting that EphB2/B3-ephrinB1/B2 signaling is necessary for an adequate immune response. In these experimental models, reduced EAE has been related to defective Th1 and Th17 cells [19] and defects in EphB6 course with compromised functions (i.e., lymphokine secretion, T cell proliferation and delayed-type skin hypersensitivity) but with normal thymic T-cell subpopulations [17]. On the contrary, conditioned EphB4 deletion in TECs does not result in functional or phenotypical immune changes [44].

In any case, although the phenotypic differences between the three experimental groups analyzed herein are not too important, some results deserve special attention, prompting speculation about the different responses observed in WT and EphB-deficient mice to collagen immunization, whether they promote (or not) the appearance of arthritic lesions:

- In non-immunized mice, the lymphoid cell subsets identified in the draining ILN exhibit a tendency to generate less reactive T cells, with higher proportions of Treg cells in EphB3^−/−^ mice than in non-immunized WT ones, or higher proportions of IL4-producing cells in both EphB2 and EphB3 mutants than in WT mice (see Fig. 4). This may indirectly suggest that these T-cell subsets could be important for governing the response to type II collagen.
- On day 34 postimmunization, WT mice developing arthritis show high proportions of Th17 cells compared to those found in immunized mutants, but the increase is similar to that shown by healthy WT mice, although WT non-CIA exhibit significantly higher proportions of IL4-producing cells (see Fig. 5). These results suggest that IL4 could be the key cytokine that determines the occurrence or not of CIA in our experimental models.
- From day 34 onward, the number of sick EphB3^−/−^ mice remains unchanged suggesting that these animals contain less reactive T cells to chicken type II collagen than WT mice. In addition, at these last stages, less activated cells would leave the WT ILN because both the proportions of IL2- and IL17-producing cells remain high, although there are also high values of IL4-producing cells and Treg cells. In agreement with our results, both Th1 and Th17 cells are induced in CIA, although Th17 cells appear to play the major pathological role [45]. In fact, Treg cells, Th1 cells and Th17 cells are key T cell populations involved in joint inflammation [46]. On the other hand, IL4 cytokine and Treg cells would be regulators of the pro-inflammatory activity of Th1 and Th17 cells. In fact, IL4 plays a protective role in CIA [25]. IL4 administration reduces clinical parameters of RA [47, 48] and its overexpression is moderately effective at reducing bone erosion and joint damage [49]. On the contrary, Treg cells described in CIA joints, synovial fluid and draining lymph nodes [50, 51] could have compromised activity [52, 53], although the Treg/Th17 cell balance is considered key for disease progression [24].
- One of the most remarkable findings of our study is that, despite the fact that EphB2^−/−^ mice were not able to develop arthritis, they do not exhibit important differences in the proportions of studied T-cell subsets when compared to other experimental groups, suggesting that the absence of sick EphB2^−/−^ animals could be related more to an altered functional capacity than to a specific T-cell phenotype. In this regard, altered peripheral tolerance mechanisms have been proposed to be involved in the disease process [43].

The analysis of both disease incidence and the T-cell subsets involved indicates that, while the behavior of WT mice in terms of immune response to chicken type II collagen is similar to that repeatedly published by other authors, emphasizing the role of Th17 cells, IL4-producing cells and Treg cells in the development of RA [25, 46], the mice deficient in EphB2 or EphB3 seem to exhibit altered functional properties that affect their reactivity to type II collagen or other immune stimuli, or their migratory capacities. Both issues have been reported in mutants deficient in other Ephs or ephrins that exhibit a low incidence of autoimmune disease [19, 21, 43], or reduced immune reactivity against different stimuli [17, 20, 54].

In addition, the functional alterations exhibited by EphB2- and EphB3-deficient mice are different, although the final result is a null or low incidence of arthritic lesions, respectively, and therefore of sick mice. According to our results, EphB2 mutants exhibit low immune reactivity, as demonstrated by the low production of antibodies or the smaller proportions of Tfh cells after immunization with type II collagen, and the reduced proliferation of ILN T cells after stimulation with anti-CD3 and anti-CD28 antibodies. However, these EphB2^−/−^ CXCR4^+^ T cells show a similar migratory capacity to CXCL12 as WT cells.

In agreement with these results, T-cells derived from ephrin-B1/ephrin-B2 double KO mice, which also show a low incidence of autoimmune diseases, exhibit less capacity than normal T cells to differentiate to Th1 cells and Th17 cells, resulting in compromised allograft rejection and anti-viral immunity [20]. The presence of autoantibodies [27] and B lymphocytes [55, 56] are, like the different T cell subsets, key elements for the development of RA. Thus, as observed in EphB3-deficient mice, type II collagen specific total IgG levels are significantly lower in ephrin-B1/ephrin-B2 double KO mice exhibiting low autoimmune reactivity than in WT, and a similar reduction in the numbers of plasma cells and Tfh cells is observed in the draining lymph nodes [22]. These authors have proposed that in these mutants, T cells are engaged in helping the B lymphocytes [22].

On the other hand, EphB3^−/−^ T cells respond like WT T cells to anti-CD3 plus anti-CD28 antibodies, producing significantly low levels of IgG against type II collagen and increasing their relative proportion of ILN Tfh cells after collagen administration, although their reactivity is significantly lower than that of WT mice. This low reactivity, together with a minimal capacity of migration in response to CXCL12 in transwell assays, explains the lower capacity of EphB3^−/−^ T cells to induce arthritic lesions as compared to WT mice. In fact, numerous studies have demonstrated the functional relationship of Eph/ephrin A [57–59] and B signaling [43, 60] with cell migration. Thus, ephrin-B are involved in T cell trafficking to inflamed paws in CIA [22] or to central nervous system in EAE murine models [19, 61], and double deleted expression of ephrin-B1 and ephrin-B2 in T cells make them less susceptible to EAE presumably by their impaired T cell chemotaxis [19].

In summary, the altered activation of T cells in response to type II collagen observed in EphB2^−/−^ mice would explain the null generation of sick animals. On the other hand, EphB3-deficient mice respond to type II collagen, but would be unable to migrate to joints in a sufficient number to induce a high number of arthritic lesions. Therefore, EphB-deficient thymuses appear to function immunologically well, as a consequence of a correct positive and negative selection [5] that correlates with the lack of autoreactive lymphoid infiltrates and autoantibodies. However, alterations in the functional properties of peripheral T cells, due to the lack of an accurate EphB/ephrin-B signaling, affect either antigen reactivity or cell migration to inflamed tissues, and would impede the development of arthritis.

### Supplementary Information

Below is the link to the electronic supplementary material.

Supplementary file1 (DOCX 248 KB)

## Data Availability

The data generated during the study are available from the corresponding author upon reasonable request.
