# Supplementary material for: Peripheral T-cell responses of EphB2- and EphB3-deficient mice in a model of collagen-induced arthritis
Source: Cell Mol Life Sci. 2024 Apr 1;81(1):159. doi: 10.1007/s00018-024-05197-0 (PMC10984909; doi:10.1007/s00018-024-05197-0)
Supplement: Supplementary file 1 — Supplementary file1 (DOCX 248 KB) [file 18_2024_5197_MOESM1_ESM.docx]

**Peripheral T-cell responses of EphB2- and EphB3-deficient mice in a model of collagen-induced arthritis**

Sara Montero-Herradón^1,2^*, Javier García-Ceca^1,2^*, Marta Villarejo-Torres^1^, and Agustín G Zapata^1,2^†

^1^ Department of Cell Biology, Faculty of Biological Sciences. Complutense University of Madrid.

^2^ Health Research Institute, Hospital 12 de Octubre (imas12).

* These authors contribute equally to the study

† Corresponding author: Agustín G Zapata; Department of Cell Biology, Faculty of Biological Sciences. Complutense University of Madrid, 28040 Madrid, Spain. zapata@ucm.es

ORCID: García-Ceca, J: 0000-0002-0940-455X; Montero-Herradón, S: 0000-0003-2004-8987; Zapata, AG: 0000-0003-0576-2672

**Online Resource 1.** Flow cytometry antibodies

| **Antibody** | **Fluorochrome** | **Clone** | **Company** |
| --- | --- | --- | --- |
| anti-CD4 | PerCP, APC, FITC | GK1.5 | Biolegend |
| anti-CD8α | APC, PE | 53-6.7 | Biolegend |
| anti-TCRβ | FITC | H57-597 | Biolegend |
| anti-CD3ɛ | FITC | 145-82C11 | Biolegend |
| anti-CD19 | FITC, APC | 1D3/CD19 | Biolegend |
| anti-CD49b | FITC | DX5 | Biolegend |
| anti-CD11c | PE | N418 | Biolegend |
| anti-CD317 (PDCA1) | PE | 129c1 | Biolegend |
| anti-CD172a (Sirpα) | APC | P84 | Biolegend |
| anti-CD26 (Dpp4) | FITC | H194-112 | Biolegend |
| anti-CD45 | PerCP | 30-F11 | Biolegend |
| anti-podoplanin (gp38) | PE | 8.1.1 | Biolegend |
| antiCD140a (PDGFRα) | APC | APA5 | Biolegend |
| antiCD140b (PDGFRβ) | APC | APB5 | Biolegend |
| anti-EpCAM (CD326) | APCCy7 | G8.8 | Biolegend |
| anti-CD31 | PacificBlue | 390 | Biolegend |
| anti-CD25 | PerCP | PC61 | Biolegend |
| anti-CD62L | PerCP | MEL-14 | Biolegend |
| anti-CXCR4 | PE | 2B11 | eBioscience |
| anti-CD185 (CXCR5) | PE | L138D7 | Biolegend |
| anti-CD279 (PD1) | FITC | 29F.1A12 | Biolegend |
| anti-IFNγ | PE | XMG 1.2 | Biolegend |
| anti-IL2 | APC | JES6-5H4 | Biolegend |
| anti-IL4 | PE | 11B11 | Biolegend |
| anti-IL17 | APC | TC11-18H10.1 | Biolegend |
| anti-FoxP3 | PE | FJK-16s | eBioscience |

**Online Resource 2.** Gating strategy for analysing inguinal lymph node lymphocytes by flow cytometry.


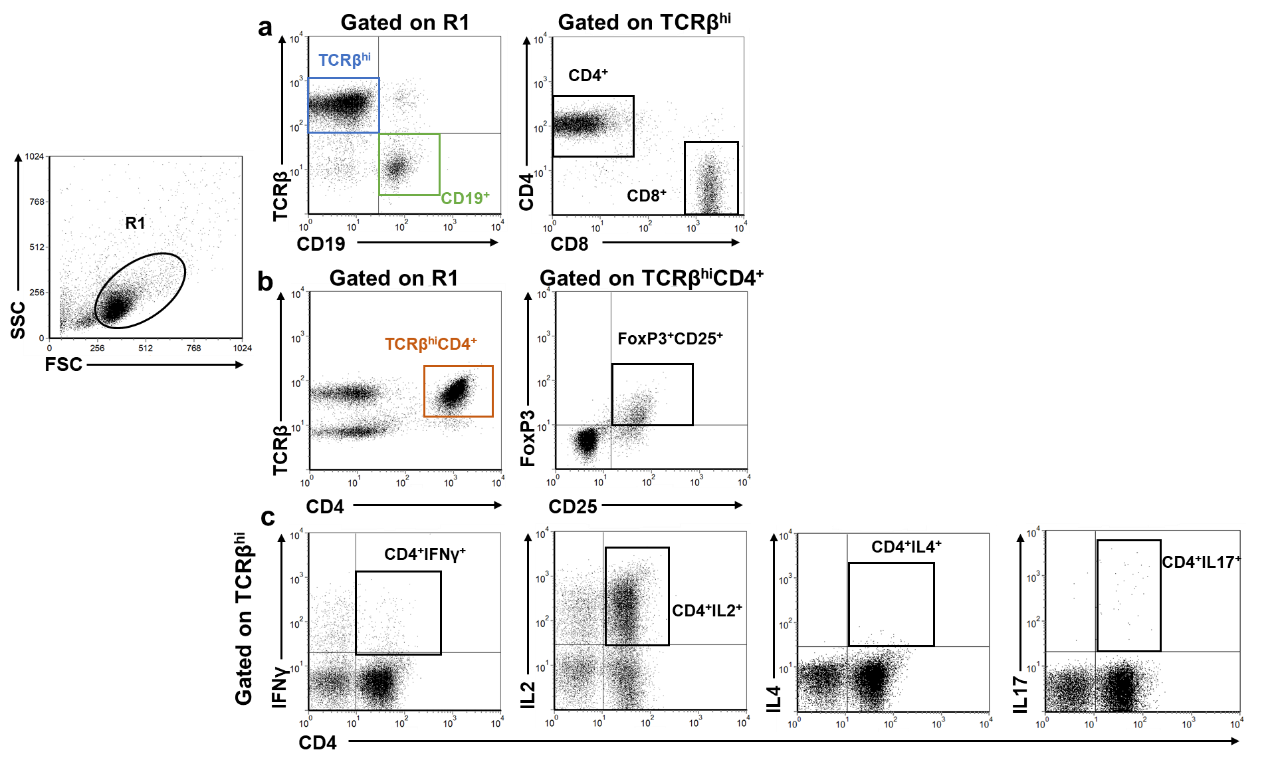


Total lymphocyte subset (R1) was identified according to FSC and SSC parameters. (a) Total T-cells (TCRβ^hi^CD19^-^ cells) and B cells (TCRβ^-^CD19^+^ cells) were identified by gating on R1 according to TCRβ or CD19 marker expression. The proportions of T-cell subsets CD4^+^ (CD4^+^CD8^-^ cells) and CD8^+^ (CD4^-^CD8^+^ cells) were determined within the TCRβ^hi^ cell population. (b) CD4^+^ regulatory T-cell subset (Treg) corresponds to FoxP3^+^CD25^+^ cells gated on TCRβ^hi^CD4^+^ cells and (c) different CD4^+^ T-cell subsets including Th1 (CD4^+^IFNɣ^+^ cells and CD4^+^IL2^+^ cells), Th2 (CD4^+^IL4^+^ cells) and Th17 (CD4^+^IL17^+^ cells) were determined by gating on TCRβ^hi^ cells.
